# Diversity of Structural, Dynamic, and Environmental Effects Explain a Distinctive Functional Role of Transmembrane Domains in the Insulin Receptor Subfamily

**DOI:** 10.3390/ijms24043906

**Published:** 2023-02-15

**Authors:** Yaroslav V. Bershatsky, Andrey S. Kuznetsov, Aisha R. Idiatullina, Olga V. Bocharova, Sofya M. Dolotova, Alina A. Gavrilenkova, Oxana V. Serova, Igor E. Deyev, Tatiana V. Rakitina, Olga T. Zangieva, Konstantin V. Pavlov, Oleg V. Batishchev, Vladimir V. Britikov, Sergey A. Usanov, Alexander S. Arseniev, Roman G. Efremov, Eduard V. Bocharov

**Affiliations:** 1 Shemyakin–Ovchinnikov Institute of Bioorganic Chemistry, Russian Academy of Sciences, Moscow 117997, Russia; 2 Moscow Institute of Physics and Technology, Dolgoprudny 141700, Russia; 3 Federal State Budgetary Institution “National Medical and Surgical Center named after N.I. Pirogov”, Moscow 105203, Russia; 4 Federal Clinical Center of Physical-Chemical Medicine of FMBA, Moscow 119435, Russia; 5 Frumkin Institute of Physical Chemistry and Electrochemistry of Russian Academy of Sciences, Moscow 119071, Russia; 6 Institute of Bioorganic Chemistry, National Academy of Sciences of Belarus, 220141 Minsk, Belarus; 7 Department of Applied Mathematics, National Research University Higher School of Economics, Moscow 101000, Russia

**Keywords:** receptor tyrosine kinases, insulin receptor subfamily, transmembrane domain, structural-dynamical properties, protein-lipid interactions, NMR, molecular dynamics

## Abstract

Human InsR, IGF1R, and IRR receptor tyrosine kinases (RTK) of the insulin receptor subfamily play an important role in signaling pathways for a wide range of physiological processes and are directly associated with many pathologies, including neurodegenerative diseases. The disulfide-linked dimeric structure of these receptors is unique among RTKs. Sharing high sequence and structure homology, the receptors differ dramatically in their localization, expression, and functions. In this work, using high-resolution NMR spectroscopy supported by atomistic computer modeling, conformational variability of the transmembrane domains and their interactions with surrounding lipids were found to differ significantly between representatives of the subfamily. Therefore, we suggest that the heterogeneous and highly dynamic membrane environment should be taken into account in the observed diversity of the structural/dynamic organization and mechanisms of activation of InsR, IGF1R, and IRR receptors. This membrane-mediated control of receptor signaling offers an attractive prospect for the development of new targeted therapies for diseases associated with dysfunction of insulin subfamily receptors.

## 1. Introduction

Receptor tyrosine kinases (RTKs) are ubiquitous receptors in the human organism. The human RTKs family contains 58 receptors divided into 20 subfamilies. The insulin receptor subfamily contains three highly homologous receptors: the insulin receptor (InsR), the insulin-like growth factor-1 receptor (IGF1R), and an orphan insulin receptor-related receptor (IRR) [1]. These proteins are involved in fundamental physiological processes such as growth, division, differentiation, and survival. Abnormal functioning of the receptors has been associated with a wide range of human pathologies including diabetes, cancer, and neurodegenerative disorders such as Alzheimer’s disease [2,3,4]. Members of the insulin receptor subfamily share more than 70% sequence homology [5,6,7], however, they differ dramatically in terms of localization, expression, and functional roles. IGF1R regulates cell growth, proliferation, and differentiation [8]. Besides cell signaling, InsR plays a key role in the metabolism of lipids, proteins, and carbohydrates as well as in the maintenance of glucose homeostasis [3]. Both receptors can be activated by peptide hormones (insulin, IGF1, and IGF2) and are widely expressed in diverse tissues of the organism. In contrast, expression of IRR is apparently highly restricted to the kidney, stomach, and pancreas. Rather than having a peptide ligand, IRR is activated by alkaline pH medium. IRR functions as an extracellular alkaline sensor and manages metabolic bicarbonate excretion [9]. Moreover, it is involved in the regulation of insulin secretion in pancreatic β-cells [10].

The insulin receptor subfamily has a unique structure among RTKs. Each receptor monomer consists of two disulfide-bonded polypeptide chains, α and β. On the cell surface, the receptors exclusively exist as preformed covalently bound dimers (αβ)_2_ [11]. Molecular architecture of the receptor dimer includes an extracellular ligand binding domain known as disulfide-linked dimeric ectodomain (ECD), two intracellular tyrosine kinase domains (KD), and two single-helix transmembrane domains (TMD) connected with other domains via flexible juxtamembrane regions (JM) [12]. Binding of ligands to the dimeric ECD leads to allosteric conformational rearrangement of the whole receptor molecule, resulting in asymmetric dimerization of kinase domains. This subsequently triggers downstream signaling pathways. Over recent years, much structural and biochemical knowledge has been obtained for isolated domains, including high-resolution structures of ECD and the kinase domain, of members of the insulin receptor subfamily [13,14,15,16,17,18]. Although no high-resolution structure of the full-length receptors of the InsR subfamily (as well as of any RTK) has yet been reported, the overall receptor conformations in different functional states have been recently described. In the absence of ligands, the InsR and IGF1R ectodomains adopt a symmetric Λ-shape (or lambda-shape) conformation [13,14,16] with spatially separated TMDs [19]. Upon ligand binding, IGF1R-ECD and InsR-ECD adopt Γ- and T-shape conformations [15,18,20,21]. These states are characterized by small distances between the fibronectin domains and TMDs of the receptors. As it would be expected from the high sequence homology within the subfamily, IRR-ECD undergoes similar conformational transitions [22]. An additional “drop-like” shape of IRR-ECD with a short distance between the two membrane-proximal FnIII-3 domains was also described [17,22].

To date, the structural–dynamic information concerning TM segments of the insulin receptor subfamily remains scarce. Functionally, TMD anchors the receptors in the plasma membrane and participates in proper transmission of the ligand-binding signal. Moreover, substitution of InsR-TMD by TMD of glycophorin A (GpA) markedly suppresses insulin signaling [23]. In addition, a single amino acid substitution in the TMD of InsR and IGF1R is enough to cause basal activation of the receptors [24,25]. Although TMDs of the receptors do not interact with ligands directly, they play an important role in the molecular mechanism of receptor activation. In contrast to well-studied ECDs of the receptors, there is only one high-resolution structure for InsR-TMD obtained by NMR spectroscopy [26]. In addition, MD simulations of conformational behavior of InsR-TMD and IGF1R-TMD in lipid membrane have been reported [27,28,29]. Due to the lack of experimental information, the process of signal transduction across the plasma membrane via TMD remains poorly understood at the molecular scale.

In this work, we aim to compare the structures and dynamics of TMDs of all three representatives—InsR, IGF1R, and IRR—from the subfamily. In particular, we conducted structural studies of their TMDs in membrane-mimicking micelles using NMR spectroscopy. Furthermore, we probed conformational variability of the three TMDs using molecular dynamics (MD) simulations of the NMR-derived structures in different lipid bilayers. Each TMD model has some distinguishing features. Both InsR and IGF1R TMD structures form α-helices with a kink at residues Pro^952^ and Pro^941^, respectively. Moreover, IGF1R-TMD has a higher flexibility in comparison with InsR-TMD. A distinctive feature of IRR-TMD is two short α-helices connected through a flexible linker Thr^925^AlaThrPro^928^. Thus, the conformational variability of TMDs and their interactions with surrounding lipids were found to differ significantly between these three representatives, suggesting the existence of membrane-mediated control of the receptor activation and functioning within the subfamily. Moreover, the observed TMD flexibly of all three proteins suggests a unique molecular mechanism of the receptor activation distinct in some details from other RTKs.

## 2. Results

### 2.1. Conformational Flexibility of TMD in IRR Differs from That in InsR and IGF1R: NMR Data

In order to characterize and compare the TMD structural–dynamic properties within the insulin receptor subfamily, we obtained the ^13^C,^15^N-labeled peptides InsRtm, IGF1Rtm, and IRRtm, containing the hydrophobic TM segments flanked by short polar N- and C-terminal regions corresponding receptor fragments InsR_951–982_, IGF1R_931–962_, and IRR_917–948_, respectively. The peptides were solubilized in the aqueous suspension of dodecylphosphocholine (DPC) micelles at a detergent/peptide (D/P) molar ratio of 160 to shift the predominant dynamic equilibrium state of the peptides into monomer form [28]. The prepared samples were investigated by means of a conventional ^1^H/^13^C/^15^N-heteronuclear NMR technique at 313 K, pH 6.7 [30,31].

**Figure 1:**
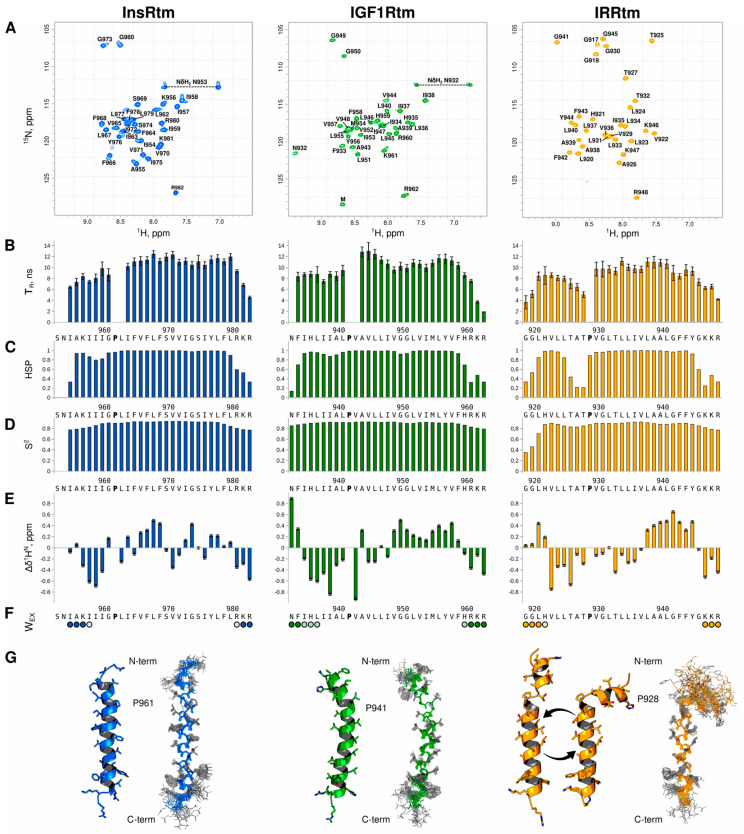
Comparison of structural–dynamic properties of InsRtm, IGF1Rtm, and IRRtm revealed by NMR studies in a membrane-mimicking environment. (**A**) ^1^H/^15^N-HSQC NMR spectra of TMDs (InsRtm in blue, IGF1Rtm in green, IRRtm in yellow) embedded into DPC micelles at D/P of 160, 313 K, and pH 6.7. The ^1^H/^15^N backbone and side-chain resonance assignments are marked. (**B**) The effective rotational correlation times, τ_R_, estimated using ^15^N CSA/dipolar cross-correlated transverse relaxation experiment [34], suggest local mobility of the TMD amide groups. Uncertainties are shown by bars. (**C**) and (**D**) The helical structure probabilities (HSP) and local order parameters S^2^, respectively, calculated based on the backbone chemical shifts [35], are indicative of helical structure distribution for TMDs and its stability along the peptide sequence. (**E**) The ^1^H^N^ secondary chemical shifts, Δδ^1^H^N^, are related to a variation of the length of the hydrogen bond, in which amide proton participates [36]; the local increase in Δδ^1^H^N^ is specified in the shortening of the given hydrogen bond, and vice versa. (**F**) Accessibility of TMD backbone amide protons to water (W_ex_) is assessed by detection of strong (solid circles) and weak (open circles) cross-peaks observed in CLEANEX spectrum [37]. (**G**) Ribbon diagrams (left) and an ensemble of 20 NMR-derived structures (right) of InsRtm (PDB 7PHT), IGF1Rtm (PDB 7PH8), and IRRtm (PDB 7PL4) embedded into DPC micelles. Bonds between heavy atoms are shown.

According to the characteristic chemical shift distribution observed in the heteronuclear NMR ^1^H/^15^N-HSQC spectra (Figure 1A), the overall fold of the TMDs in the micelles is a typical α-helical structure [32] that is consistent with a pattern of nuclear Overhauser effect (NOE) contacts identified in the NMR spectra (see Supplementary Figure S1). The structure of each TMD was calculated with a high-resolution level with CYANA [33] based on obtained ^1^H–^1^H NOE connectivities and torsion angle restraints. An overview of the structural statistics for the ensemble of 20 structures of the TMDs with the lowest target function is represented in Supplementary Table S1. Resultant ^1^H, ^13^C, ^15^N chemical shift assignments, NMR-derived constraints, and atomic coordinates of InsRtm, IGF1Rtm, and IRRtm have been deposited in the Protein Data Bank under the accession codes (PDB ID) 7PHT, 7PH8, and 7PL4, respectively.

The calculated TMD structures of IGF1Rtm and InsRtm reveal significant differences as compared to IRRtm. Thus, InsRtm and IGF1Rtm consist of single α-helix I^954^–R^980^ and F^933^–R^960^, respectively, with a slight bend near the intramembrane proline residues P^961^ and P^941^. In contrast, IRRtm includes two helical regions: G^919^–T^925^ and V^929^–K^946^ (Figure 1), connected via a flexible loop A^926^TP^928^.

In all cases, the intramembrane prolines destabilize the TMD helices, causing a kink that structurally separates the helices into the N- (n-term) and C-terminal (c-term) parts. P^928^ of IRRtm has the most pronounced eroding effect on the helix and splits it into two parts (Figure 1G, yellow). The ensemble of 20 IRRtm structures can be subdivided in two main groups of conformations: I-state and Γ-state. I-state or straight conformation is characterized by the distance between the n-term G^919^ and the c-term K^946^ of ~4 nm and the angle between the n- and c-term helices of ~26°. Γ-state or bent conformation is characterized by the distance between n-term G^919^ and c-term K^946^ of ~33 Å and the angle between the helices of ~70°. InsRtm (Figure 1G, blue) and IGF1Rtm (Figure 1G, green) prolines exert comparable influence on the geometry of the helices; in both cases, TMDs remain intact. InsRtm proline (P^961^) bends the TMD helix with the angle of ~26° between n- and c-term helical part A^955^–G^960^ and P^961^–L^979^ and the distance of ~40 Å between A^955^ and R^980^. IGF1Rtm helix has two irregularities at P^941^ and G^949^G^950^. The bending angle of ~30° is observed between the n-term and the central helical part F^933^–L^940^ and P^941^–V^948^ followed by a ~10° kink at G^949^G^950^. Nevertheless, the distance between n-term F^933^ and c-term R^960^ of IGF1Rtm is the same 40 Å as for the other TMD structures, with the exception of the bent state of IRRtm.

These distinct conformational properties of IRRtm are supported by additional structural data based on chemical shifts. Analysis of the helical secondary structure probabilities (HSP) and local order parameters S^2^ derived from ^1^H, ^13^C, and ^15^N chemical shifts revealed similar differences between the TMDs. While the internal parts of InsRtm and IGF1Rtm have strong helical conformation with slight weakening of helicity before the intramembrane proline residue (Figure 1C,D), IRRtm has dramatically lower HSP and S^2^ values, which indicate a higher mobility in this TMD part. The effective rotational correlation times (τ_R_) estimated from ^15^N CSA/dipolar cross-correlated transverse relaxation of the TMD amide groups are in agreement with this assumption (Figure 1B). Moreover, in all three cases, the intramembrane proline residues divide the TMDs into two regions: a relatively mobile n-term region and a stable c-term region. InsRtm and IGF1Rtm have the average τ_R_ higher than that of IRRtm. The average τ_R_ values of InsRtm and IGF1Rtm equals 8.3 ns and 8.6 ns for the regions before proline, and 10.7 ns and 11.2 ns for the regions after proline, respectively. IRRtm is the least stable TMD out of the three fragments. It has an average τ_R_ of 7.9 ns for the region before proline and only 9.6 ns for the region after proline. Furthermore, we observed a decrease in local τ_R_ of T^925^AT^927^ below 6 ns. It should be noted that the n-term part of each TMD has a negative magnitude of ^1^H^N^ secondary chemical shifts, Δδ^1^H^N^ (Figure 1E), which indicate increased HN⋯OC H-bond lengths, supporting the observed decrease in local τ_R_ values due to some destabilization of the TMD helices of InsRtm, IGF1Rtm, and IRRtm before the intramembrane prolines. In addition, according to the τ_R_ values decreasing to ~4–8 ns and the water-exchange data, the terminal parts of the TMDs are flexible (Figure 1B) and accessible to water (Figure 1F).

### 2.2. TMDs of InsR, IGF1R and IRR Dynamically Adapt to Different Lipid Bilayers: MD Simulations

In order to study conformational behavior in different membrane environments, the obtained NMR-derived structures of the TMDs from the insulin receptor subfamily were relaxed by MD in two explicit lipid bilayers consisting of POPC and DMPC surrounded by water molecules. POPC and DMPC membranes have different properties, such as acyl chain ordering and membrane thickness; POPC forms a bilayer with hydrophobic thickness of 2.9 nm while DMPC is thinner by approximately 0.14 nm [38]. Starting with the experimental structures, we carried out short MD equilibrations with subsequent 200-ns unconstrained MD simulations.

**Figure 2:**
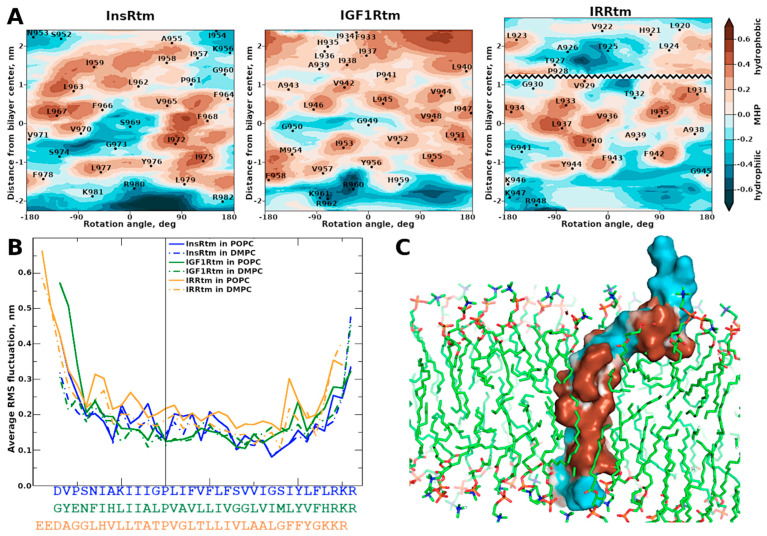
Hydrophobic properties and dynamics of NMR-derived structures of InsRtm, IGF1Rtm, and IRRtm in explicit lipid bilayers. (**A**) Two-dimensional MHP maps of TMD helices: cylindrical projections of the surface MHP [41,42] averaged over MD trajectories in the POPC bilayer. X- and Y-axes correspond to the rotation angle around the helix axis and the shift along it, respectively. Hydrophobic surface regions are shown in brown, and hydrophilic ones—in cyan. In the case of IRRtm, the map is divided into two parts corresponding to the n- and c-term helices separated by intramembrane proline P^928^. The MHP scale is shown on the right. MHP values are given as log*p* values, where *p* is the octanol-1/water partition coefficient. (**B**) Per-residue root-mean square fluctuations (RMSF) averaged over MD trajectories for InsRtm (blue), IGF1Rtm (green), IRRtm (yellow) in POPC and DMPC bilayers. (**C**) Representative TMD structure (of IRRtm) after MD-relaxation in explicit lipid bilayer (POPC). The molecular surface of TMD is colored according to MHP values.

According to RMFS values averaged over MD trajectories for each TMD (Figure 2B), the n-term regions situated before the intramembrane prolines have increased mobility in both lipid bilayers. This agrees well with NMR-relaxation data. Remarkably, the RMFS increase is more pronounced in the thicker POPC bilayer for all TMDs. Comparison of RMSF values for TMDs also reveals increased mobility of IRRtm within the membrane, which is fully consistent with NMR measurements.

As shown in Figure 2, each TMD has a unique distribution of hydrophobic/hydrophilic regions on the helix surface. Nevertheless, the specific patterns of relatively hydrophilic regions, which may be potentially employed for the TMD dimerization within the hydrophobic membrane environment [39,40], can be identified in both N- and C-terms of all TMDs of the insulin receptor subfamily. Notably, the n-term hydrophilic regions are adjacent to the intramembrane prolines and should be partially perturbed by the helix bending that is particularly visible in the case of IRRtm.

**Figure 3:**
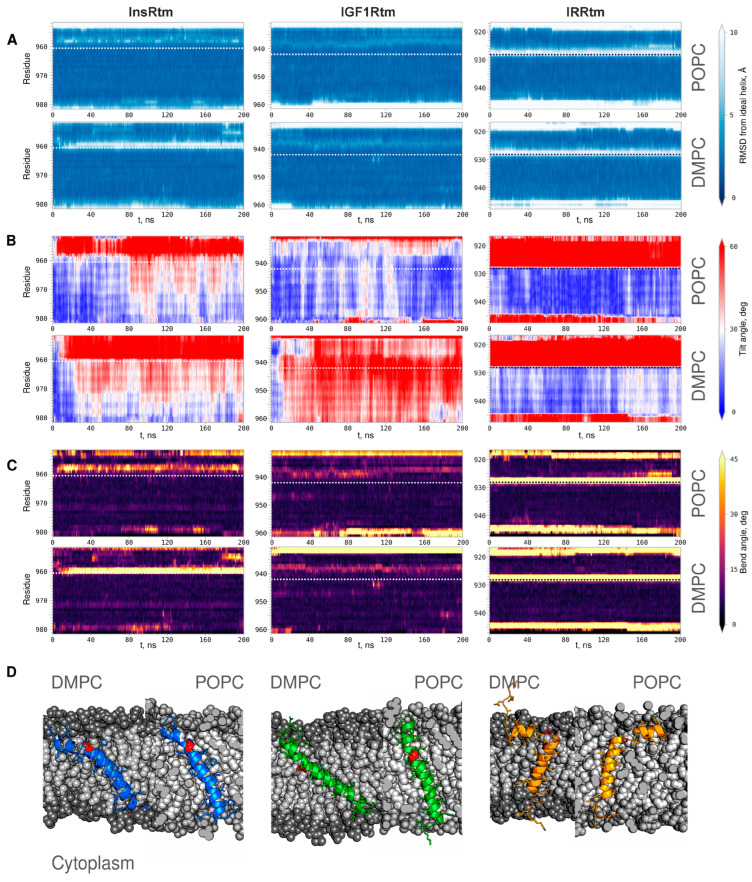
Conformational behavior of InsRtm, IGF1Rtm, and IRRtm during MD simulations in hydrated lipid bilayers composed of DMPC and POPC. Color-coded maps present time evolution of (**A**) RMSD from the ideal helix for the secondary structure, (**B**) local helix tilt angle of TMDs during MD simulations, (**C**) local helix bending of InsRtm, IGF1Rtm, and IRRtm relaxed in DMPC (bottom panels) and POPC (top panels) bilayers. On the right, the color-coding bars of the corresponding values are presented. (**D**) Representative MD snapshots of the InsRtm, IGF1Rtm, and IRRtm structures relaxed in DMPC (left) and POPC (right) bilayers (shown by gray spheres). TMD fragments are given as ribbon representation in blue (InsRtm), green (IGF1Rtm), and yellow (IRRtm); intramembrane proline residues are shown in red.

According to the structural analysis, the single-helix conformation of IGF1Rtm remains mainly intact during all MD simulation experiments. In both DMPC and POPC environments, it has small values of RMSD from the ideal helix in region I^936^IAL^940^ before intramembrane proline P^941^ (Figure 3A). This manifests as helix bending with the angle of ~25° in this TMD part, which is also observed in the NMR-derived structure of IGF1Rtm (Figure 3C). In addition, the C-terminus of the helix undergoes partial unfolding. IGF1Rtm adapts to the membrane environment changes mainly via the helix tilting: in a thin DMPC bilayer, the IGF1Rtm helix axis deviates by up to 60° from the membrane normal, while in a thicker POPC bilayer, the helix stays close to the membrane normal (Figure 3B). The final conformations of IGF1Rtm taken from the MD trace (Figure 3D) clearly demonstrate the features described above.

During all MD simulations, the intramembrane P^928^ divides IRRtm into two helices connected by a flexible linker (Figure 3A). Being buried into lipids, the short n-term helix (G^919^–T^925^) mainly lays parallel to the membrane surface in both lipid bilayers. The longer c-term helix (V^929^–K^946^) crosses the POPC and DMPC bilayers, having the tilt angle of ~15° and ~30°, respectively (Figure 3B). Moreover, due to high flexibility of the linker, the helices adopt different mutual positions, enabling TMD to adjust to the membrane thickness.

In contrast, the conformational behavior of InsRtm in MD is different from that of other subfamily members, and its structure and dynamics depend on the membrane environment used. In the POPC membrane, InsRtm presents as a single helix with a ~30° bend before intramembrane proline P^961^, similarly to IGF1Rtm (Figure 3C). Though the tilt angle of the helix N-terminal part is ~60°, most of the TMD helix is oriented close to the membrane normal (Figure 3B). Decreased thickness of the DMPC bilayer causes unwinding of the helix turn before P^961^ that results in the appearance of a flexible linker dividing InsRtm into mutually mobile n- and c-term helices N^953^–I^958^, and L^962^–R^981^ in a fashion similar to the IRRtm case (Figure 3D). Nevertheless, the flexible linker formation does not fully compensate for the change in membrane thickness, and in the DMPC bilayer, the InsRtm helix region after the proline residue is tilted more strongly than in the POPC bilayer with tilt angles of ~45° and ~30°, respectively (Figure 3B). 

## 3. Discussion

In the present NMR studies, we found that all TMDs of InsR, IGF1R, and IRR have bends before the intramembrane proline residues P^961^, P^941^, and P^928^, respectively (referred to below as the ‘P-hinge’). This leads to increased mobility of the N-terminal part of TMD helices in the micellar medium mimicking a membrane. Moreover, IRR TMD undergoes a conformational transition between the two main I- and Γ-states corresponding to the straight and bent structures of the TMD helix divided by the P-hinge into mutually mobile N- and C-term parts. MD simulations of the NMR-derived structures in two explicit lipid bilayers composed of POPC and DMPC revealed that InsR TMD embedded into thin DMPC membrane can also switch between two main states I and Γ due to unfolding of the helix turn (forming P-hinge) adjacent to the intramembrane proline. At the same time, TMD of IGF1R compensates for the decrease in membrane thickness by tilting—this is consistent with previous MD simulations [27]. Nevertheless, earlier biophysical studies [29] have shown that the TMD of IGF1R can also undergo similar switching between the straight and bent conformations, with the flexible P-hinge adjacent to the intramembrane proline. In addition, increased flexibility of the N-terminal part and the proline-induced kink of the TMD helix was also described before in NMR studies of InsR_940–988_ fragments elongated by JM regions [26]. This supports our current conclusions made for TMDs from the insulin receptor subfamily. According to our MD data, structural fluctuations of TMD helices increase in a bilayer composed of unsaturated POPC lipids having a double bond in one fatty acyl tail, which is more pronounced for flexible IRRtm (Figure 2B). Thus, different physical properties of the lipid environment can change the conformational behavior of TMDs, affecting the receptor functioning as a whole.

As noted above, the relatively hydrophilic surface regions (Figure 2A), which may serve as the potential specific patterns for TMD dimerization [39,40], can be identified in the TMD of each member of the insulin receptor subfamily. As revealed by recent structural studies of full-length receptors from this group [15,16,18,19,20,21,22], upon receptor activation, their TMD helices self-associate apparently via the N-terminal parts, as is typical for RTKs [39,40,43,44]. The N-terminal hydrophilic (polar) regions as potential dimerization motifs [39,40] adjacent to intramembrane prolines could be formed only in straight conformations of TMD helices, as demonstrated by us for IRRtm (Figure 2A,C) and previously proposed for IGF1R [29]. This implies that in the active state TMDs of the insulin receptor subfamily, members should be in straight conformation, leaving open a question about the potential functions of bent conformation and the C-terminal hydrophilic patterns of TMDs. As for the latter, they can serve as the C-terminal dimerization motifs participating in a transient dimerization of TMDs in the inactive (dormant) receptor state (similar to other RTKs [40,44,45,46,47,48]) or in intermediate steps, which can be adopted by the receptor upon activation. At the same time, other intermolecular interactions in the membrane involving these motifs cannot be excluded.

Although transmembrane signaling by type I receptors such as RTKs has not yet been fully elucidated, some molecular details of the process regulated by TMDs have been uncovered based on numerous structural, biophysical, and biochemical studies. Recently, we proposed a lipid-mediated mechanism implying that signal transduction through the membrane and allosteric regulation of type I receptors are inclusively mediated by coupled protein–protein and protein–lipid interactions [40,46]. 

**Figure 4:**
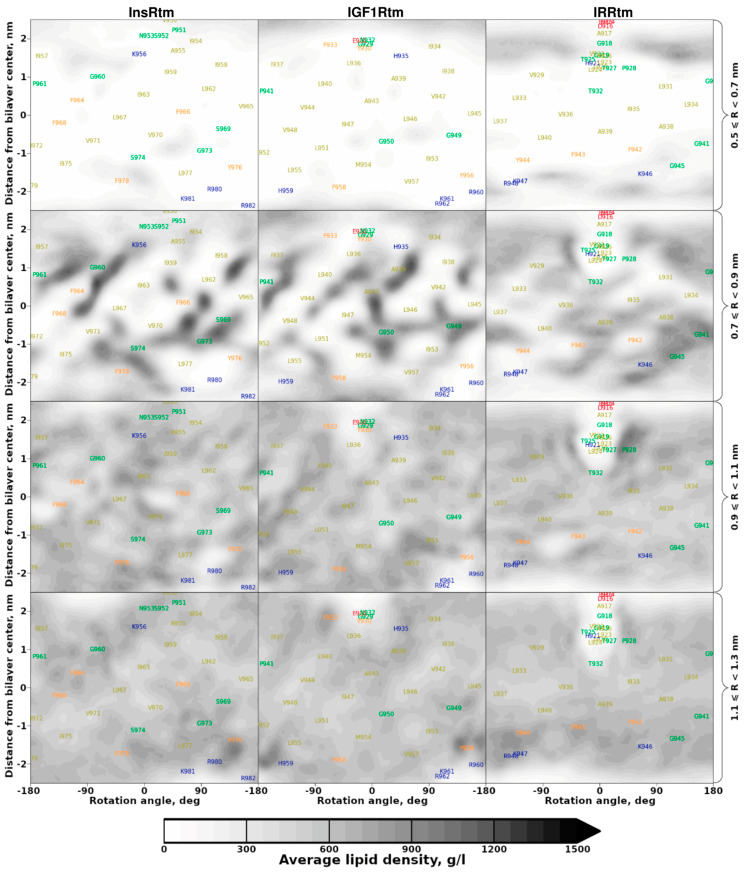
Lipid density distribution in cylindrical slices around the TMD helices. Lipid density was calculated in a cylindrical coordinate system with an axis corresponding to the TMD helix axis and averaged over MD trajectories in the POPC bilayer within the marked radius (R) range (in right). Darker areas represent larger average density values corresponding to immobilized lipids. Lower slices correspond to larger cylindrical slice radius.

To probe protein–lipid interactions, we used MD-averaged distributions of lipid density around TMDs. These data were visualized in terms of 2D density maps—projections on the surface of a cylinder built around the helix axis (Figure 4). Intriguingly, distinct lipid ‘freezing’ is observed on the TMD helix surface (at a certain distance) of InsRtm and IGF1Rtm in POPC bilayer, where both TMDs have straight-conformation. However, much less distinguishable patterns of immobilized lipid tails are observed around TMD of IRRtm. We assume that this is caused by its higher conformational mobility due to the flexible P-hinge, which permits switching between the straight and the bent structures. An additional lipid density maximum is formed under the N-terminal amphiphilic helix of IRRtm separated by the P-hinge and immersed into the lipid head group region. Thus, the flexible bent conformation of IRRtm with relatively weak lipid adsorption has an entropic preference in free energy compared to the ‘rigid’ straight conformation of InsRtm and IGF1Rtm containing strongly bound lipids on their surface. Furthermore, dimerization of TMD in the straight conformation could release a number of immobilized lipids from the TMD helix–helix interface. This, in turn, results in a certain entropy loss in the free energy. So, in their straight conformation, TMDs of the insulin receptor subfamily would prefer to self-associate, whereas a bent conformation would inhibit TMD dimerization. According to the current results, variation of the lipid bilayer properties (depending, e.g., on membrane composition and protein–lipid interactions) would affect the conformational behavior of TMDs and ultimately modulate receptor functioning. Indeed, e.g., an age-associated cholesterol reduction triggers brain insulin resistance by facilitating ligand-independent receptor activation [49], while activation of InsR and IGF1R depends critically on the structures of membrane sterols [50].

**Figure 5:**
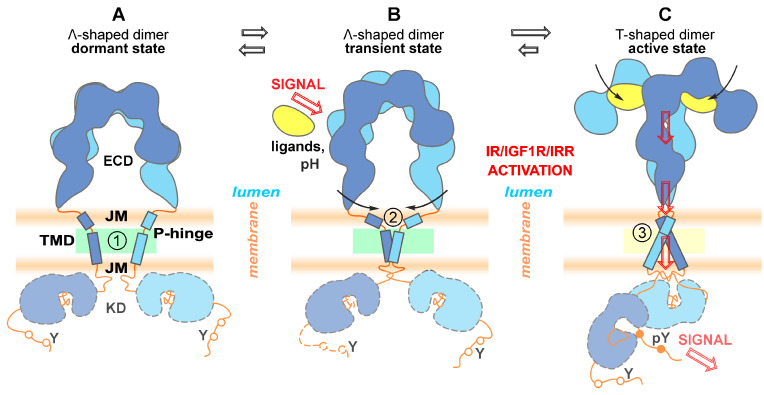
Schematic illustration of the key stages of the proposed mechanism of signal transduction by RTK of the insulin receptor subfamily. ECD—ectodomain, JM—juxtamembrane region, TMD—transmembrane domain, KD—kinase domain; P-hinge—flexible linker adjacent to intramembrane proline. Left to right: proposed structural rearrangements of the receptors inferred from structural information obtained in this work (indicated by numbers in circles) and available literature data. (**A**) Inactive state: disulfide-linked ECD is in unliganded Λ-shape conformation, separated TMD helices are bending in flexible P-hinge (marked with 1 in a circle) adjacent to intramembrane proline. The lipids surrounding TMD are relatively flexible (shown by green). (**B**) The ligand binding (in the case of InsR and IGF1R) or pH increase (in the case of IRR) induces conformational changes in ECD that bring their C-termini closer together (indicated by black arrows), resulting in transient dimerization of TMDs via C-terminal hydrophilic (polar) patterns, while the TMD helix bending in a flexible P-hinge allows to compensate for the remaining separation of ECD C-termini (marked with 2 in a circle). (**C**) Further switching of the receptor dimer accompanied by rearrangements of all domains into the active state allows phosphorylation of the target tyrosine residues (pY) followed by the stimulation of the downstream signaling cascades. The TMD helices dimerize via N-terminal hydrophilic (polar) patterns formed again by TMD straight-conformation (marked with 3 in a circle) with surrounding lipids immobilized on the helix surface (shown by yellow).

Based on the totality of experimental and calculated data obtained in this work as well as published data, we came up with the following sequences of key events leading to RTKs of the insulin receptor subfamily activation and signal transduction schematically summarized in Figure 5. We propose that there is no TMD dimer in the completely inactive receptor state (Figure 5A), but finally—after ligand binding followed by closure of the ECD C-termini (FnIII-3 subdomains)—parallel packing of TM segments results in close juxtaposition of intracellular kinase domains. Upon activation, the receptor apparently assumes an intermediate conformation (Figure 5B) with the TMD helix bending in a flexible P-hinge, allowing to compensate for the remaining spatial separation of ECD C-termini. The lipids surrounding the TMD helices are relatively flexible in the inactive and intermediate states, and immobilized on the TMD straight-helix surface in the active receptor (Figure 5C). The lipid membrane composition would influence the receptor activation, and the effect appears to be more pronounced in the case of pH-dependent activation of IRR having a large degree of P-hinge flexibility in the TMD helix. 

## 4. Materials and Methods

### 4.1. Protein Expression and Purification

The DNA sequences encoding human TMD fragments of IGF1R (residues 931–962; IGF1Rtm), InsR (residues 952–982; InsRtm), and IRR (residues 916–948; IRRtm) were synthesized from six overlapping synthetic oligonucleotides by PCR and then cloned into pGEMEX-1 vector. The genes were under the control of the T7 promoter. The final construct of IGF1Rtm was with the leading methionine: M-N^932^FIHLIIALPVAVLLIVGGLVIMLYVFHRKR^962^. InsRtm and IRRtm gene constructions contained N-terminal His-tag connected with target proteins by flexible GS linker MHHHHHHG-S^952^NIAKIIIGPLIFVFLFSVVIGSIYLFLRKR^982^ and MHHHHHHGS-A^917^GGLHVLLTATPVGLTLLIVLAALGFFYGKKR^948^, respectively. We use continuous-exchange cell-free expression (CECF) system based on E. coli S30 extract. The plasmids were used as DNA templates in the reactions [51] to produce the target proteins. The CECF production in the precipitate-CF mode was optimized with respect to concentrations of plasmid DNA using SDS-PAGE [28,52]. The yield of synthesized TMDs was ~1.5 mg from 1 mL of RM. The ^13^C,^15^N-labeled algal amino acid mixture was used for production of isotopic labeled proteins. The reactions were incubated with gentle shaking overnight at 34 °C. The peptide precipitate was separated from the reaction mixture by centrifugation. The pellet was solubilized in a buffer containing 0.5% lauroyl sarcosine, 50 mM Tris/HCl, pH 8.0, 50 mM NaCl, and size exclusion chromatography was carried out using the Tricorn 10/300 Superdex 200 Increase column at 0.5 mL/min flow rate. Fractions containing the peptide were combined, then the peptide was precipitated by addition of TCA and washed with cold acetone three times. The pellet was solubilized in 1:1 (*v/v*) trifluoroethanol–water mixture and lyophilized. In order to incorporate TMDs into membrane-mimicking micelles or bicelles, the peptide powder was dissolved in 1:1 (*v/v*) trifluoroethanol–water mixture with the addition of n-dodecylphosphocholine-d38 (d38-DPC, 98%, CIL) at the peptide:detergent (P:D) molar ratio of 1:160 [28]. The mixtures were lyophilized overnight and re-dissolved in 270 μL of water buffer solution containing 25 mM sodium phosphate, 0.3 mM NaN_3_, and 5% D_2_O (*v/v*), pH 6.7.

### 4.2. NMR Spectroscopy and Structure Calculation

NMR spectra were acquired at 313 K on 600 and 800 MHz Bruker AVANCE III spectrometers equipped with triple-resonance with H/C/N triple resonance Z-gradient cryoprobe. The ^1^H/^13^C/^15^N backbone resonances of TMDs were assigned with the CARA software [53] using the following two- and three-dimensional heteronuclear NMR experiments [30]: ^1^H/^15^N-HSQC, ^1^H/^13^C-HSQC, ^1^H/^13^C-HSQC-CT (constant time version with evolution period of 28.6 ms), ^1^H/^15^N-TROSY, ^1^H/^13^C/^15^N-HNCO, ^1^H/^13^C/^15^N-HNCA, ^1^H/^13^C/^15^N-HN(CO)CA, ^1^H/^13^C-HCCH-TOCSY (with the mixing time of 17 ms), ^1^H/^13^C/^15^N-HNCACB,^15^N-edited TOCSY-, and NOESY-HSQC with the mixing time of 80 ms. The BEST-TROSY version [54] of the triple resonance experiments was used, and the spectra were recorded with non-uniform sampling of indirect dimensions and processed using the qMDD software [55]. The spatial structure of the TMD monomers was calculated with the CYANA program [33] based on ^1^H–^1^H NOE connectivities and torsion angle restraints. Torsion angle restraints, the helical secondary structure probabilities (HSP), and local order parameters S^2^ were obtained from the secondary structure probabilities estimated from the ^1^H, ^13^C, and ^15^N chemical shift values using the web-based program TALOS-N [35]. Intramolecular ^1^H–^1^H NOE connectivities were obtained with the CARA software through the analysis of the three-dimensional ^15^N-edited NOESY-HSQC spectrum. In order to characterize the intramolecular dynamics of the TMD fragments, the effective rotation correlation times τ_R_ were estimated for individual amide groups of the fragments based on ^15^N CSA/dipolar cross-correlated transverse relaxation experiment acquired in interleaved fashion for the reference and attenuated spectra using a 2D ^1^H/^15^N-ct-TROSY-HSQC-based pulse sequence [34] with the constant period of 26.9 ms and the relaxation period of 10.8 ms. The rotational correlation times τ_R_ was calculated as the ratio of peak intensities of the reference and attenuated spectra; the uncertainties were estimated from the noise level. The water accessibility of the TMDs residues was analyzed by chemical exchange of the amide protons with water directly detected by CLEANEX [37] experiment. The ^1^H^N^ secondary chemical shifts, Δδ^1^H^N^, of the TMDs residues was calculated as the difference between actual chemical shift and typical random-coil chemical shift for a given residue [36].

### 4.3. Molecular Dynamics in Explicit Lipid Bilayer

MD simulations and data processing were carried out using the GROMACS package version 2019.4 or higher. All-atom Amber14SB protein parameters with S-lipids and TIP3P water were used to describe intermolecular interactions together with PME electrostatics and Van der Waals cut-off at 1.5 nm. To estimate stability of all the systems, they were subjected to energy minimization, MD equilibration with restrained protein, and unrestrained 200-ns MD simulations. Each TMD fragment was inserted into a pre-equilibrated hydrated palmitoyloleoylphosphatidylcholine (POPC) or dimyristoylphosphatidylcholine (DMPC) bilayer with a conformation derived from NMR data and perpendicular orientation of the helix axis with respect to the membrane plane. Relaxation was carried out by 9-ns MD simulation started from fully “frozen” protein with stepwise unfreezing of terminal amino acids as follows: First, we used constraints imposed on all protein atoms and equilibrated the hydrated lipid bilayer for 3 ns at the constant temperature of 315 K. Then, we allowed sidechains and terminal protein residues to fit to the environment during 6-ns MD. Finally, production MD runs were performed without restraints. The structure stability was estimated in terms of the root-mean-square deviation (RMSD) of coordinates of all (or only backbone) protein atoms from the starting NMR structure, protein secondary structure change, and the tilt angle between the helix axis and the membrane normal. Molecular flexibility was evaluated as per-residue root-mean-square fluctuation (RMSF) averaged over the trajectory. Molecular hydrophobic–hydrophilic properties on the helix surface were estimated using the molecular hydrophobicity potential (MHP) approach, as described previously [41,42]. Protein–lipid contacts were determined based on the average lipid density analysis based on MD trajectories. A helical fragment of each peptide (after proline residue) was selected to determine the peptide axis, and cylindrical slices were used. In the case of IRR, an additional N-terminal fragment (before proline) was analyzed as an individual helix.

## 5. Conclusions

In summary, we can conclude that the TMD sequences of all three representatives of the insulin receptor subfamily encode information about their conformational lability, which is largely determined by the intramembrane proline residue and its neighboring residues. Under the conditions of a real lipid membrane, this is expressed in the distinct features of the structural–dynamic behavior of these TMDs. It is important to note that the extent of the conformational changes undergone differs for the three proteins and, in addition, it depends on the properties of the surrounding water–lipid media. Variation of these parameters allows each of the representatives of the signaling receptor subfamily to respond sensitively to activating stimuli (ligand binding, pH changes) and the current state of the local membrane environment. This, in turn, can provide the receptors with a certain “margin of safety” necessary for the stable and robust operation of these important cell signaling systems. On the other hand, it gives them a certain flexibility, helping to synchronize with their neighbors in the “signal orchestra”—the surrounding membrane receptors. This membrane-mediated control of signaling of the insulin family receptors would also influence receptor dysfunction upon pathological conditions, which should be taken into account when developing new targeted therapies for human diseases, including neurodegenerative ones. 

## Data Availability

The assigned chemical shifts were deposited into Biological Magnetic Resonance Bank (BMRB, https://bmrb.io/) under the accession codes 34657, 34656, and 34662 for InsR, IGF1R, and IRR, respectively. The final NMR ensemble was deposited into Protein Data Bank (PDB, https://www.rcsb.org/) under the accession codes 7PHT, 7PH8, and 7PL4 for InsR, IGF1R, and IRR, respectively.

## Supplementary Materials

The supporting information can be downloaded at: https://www.mdpi.com/article/10.3390/ijms24043906/s1.

## References

[1] Lemmon M.A., Schlessinger J. (2010). Cell Signaling by Receptor Tyrosine Kinases. Cell.

[2] Frasca F., Pandini G., Sciacca L., Pezzino V., Squatrito S., Belfiore A., Vigneri R. (2008). The Role of Insulin Receptors and IGF-I Receptors in Cancer and Other Diseases. Arch. Physiol. Biochem..

[3] Saltiel A.R., Kahn C.R. (2001). Insulin Signalling and the Regulation of Glucose and Lipid Metabolism. Nature.

[4] Craft S. (2012). Insulin Resistance and AD—Extending the Translational. Path. Nat. Rev. Neurol..

[5] Ebina Y., Ellis L., Jarnagin K., Edery M., Graf L., Clauser E., Ou J., Masiarz F., Kan Y.W., Goldfine I.D. (1985). The Human Insulin Receptor CDNA: The Structural Basis for Hormone-Activated Transmembrane Signalling. Cell.

[6] Ullrich A., Gray A., Tam A.W., Yang-Feng T., Tsubokawa M., Collins C., Henzel W., Bon T.L., Kathuria S., Chen E. (1986). Insulin-like Growth Factor I Receptor Primary Structure: Comparison with Insulin Receptor Suggests Structural Determinants That Define Functional Specificity. EMBO J..

[7] Tsujimoto K., Tsuji N., Ozaki K., Ohta M., Itoh N. (1995). Insulin Receptor-Related Receptor Messenger Ribonucleic Acid in the Stomach Is Focally Expressed in the Enterochromaffin-like Cells. Endocrinology.

[8] Siddle K. (2011). Signalling by Insulin and IGF Receptors: Supporting Acts and New Players. J. Mol. Endocrinol..

[9] Deyev I.E., Sohet F., Vassilenko K.P., Serova O.V., Popova N.V., Zozulya S.A., Burova E.B., Houillier P., Rzhevsky D.I., Berchatova A.A. (2011). Insulin Receptor-Related Receptor as an Extracellular Alkali Sensor. Cell Metab..

[10] Yamaoka M., Terabayashi T., Nishioka T., Kaibuchi K., Ishikawa T., Ishizaki T., Kimura T. (2019). IRR Is Involved in Glucose-Induced Endocytosis after Insulin Secretion. J. Pharmacol. Sci..

[11] Hedo J.A., Kahn C.R., Hayashi M., Yamada K.M., Kasuga M. (1983). Biosynthesis and Glycosylation of the Insulin Receptor. Evidence for a Single Polypeptide Precursor of the Two Major Subunits. J. Biol. Chem..

[12] Ullrich A., Bell J.R., Chen E.Y., Herrera R., Petruzzelli L.M., Dull T.J., Gray A., Coussens L., Liao Y.-C., Tsubokawa M. (1985). Human Insulin Receptor and Its Relationship to the Tyrosine Kinase Family of Oncogenes. Nature.

[13] McKern N.M., Lawrence M.C., Streltsov V.A., Lou M.-Z., Adams T.E., Lovrecz G.O., Elleman T.C., Richards K.M., Bentley J.D., Pilling P.A. (2006). Structure of the Insulin Receptor Ectodomain Reveals a Folded-over Conformation. Nature.

[14] Croll T.I., Smith B.J., Margetts M.B., Whittaker J., Weiss M.A., Ward C.W., Lawrence M.C. (2016). Higher-Resolution Structure of the Human Insulin Receptor Ectodomain: Multi-Modal Inclusion of the Insert Domain. Structure.

[15] Scapin G., Dandey V.P., Zhang Z., Prosise W., Hruza A., Kelly T., Mayhood T., Strickland C., Potter C.S., Carragher B. (2018). Structure of the Insulin Receptor–Insulin Complex by Single-Particle Cryo-EM Analysis. Nature.

[16] Xu Y., Kong G.K.-W., Menting J.G., Margetts M.B., Delaine C.A., Jenkin L.M., Kiselyov V.V., De Meyts P., Forbes B.E., Lawrence M.C. (2018). How Ligand Binds to the Type 1 Insulin-like Growth Factor Receptor. Nat. Commun..

[17] Shtykova E.V., Petoukhov M.V., Mozhaev A.A., Deyev I.E., Dadinova L.A., Loshkarev N.A., Goryashchenko A.S., Bocharov E.V., Jeffries C.M., Svergun D.I. (2019). The Dimeric Ectodomain of the Alkali-Sensing Insulin Receptor–Related Receptor (EctoIRR) Has a Droplike Shape. J. Biol. Chem..

[18] Li J., Choi E., Yu H., Bai X. (2019). Structural Basis of the Activation of Type 1 Insulin-like Growth Factor Receptor. Nat. Commun..

[19] Gutmann T., Kim K.H., Grzybek M., Walz T., Coskun Ü. (2018). Visualization of Ligand-Induced Transmembrane Signaling in the Full-Length Human Insulin Receptor. J. Cell Biol..

[20] Uchikawa E., Choi E., Shang G., Yu H., Bai X. (2019). Activation Mechanism of the Insulin Receptor Revealed by Cryo-EM Structure of the Fully Liganded Receptor–Ligand Complex. eLife.

[21] Nielsen J., Brandt J., Boesen T., Hummelshøj T., Slaaby R., Schluckebier G., Nissen P. (2022). Structural Investigations of Full-Length Insulin Receptor Dynamics and Signalling. J. Mol. Biol..

[22] Batishchev O.V., Kuzmina N.V., Mozhaev A.A., Goryashchenko A.S., Mileshina E.D., Orsa A.N., Bocharov E.V., Deyev I.E., Petrenko A.G. (2021). Activity-Dependent Conformational Transitions of the Insulin Receptor–Related Receptor. J. Biol. Chem..

[23] Gardin A., Auzan C., Clauser E., Malherbe T., Aunis D., Crémel G., Hubert P. (1999). Substitution of the Insulin Receptor Transmembrane Domain with That of Glycophorin A Inhibits Insulin Action. FASEB J..

[24] Takahashi K., Yonezawa K., Nishimoto I. (1995). Insulin-like Growth Factor I Receptor Activated by a Transmembrane Mutation. J. Biol. Chem..

[25] Longo N., Shuster R.C., Griffin L.D., Langley S.D., Elsas L.J. (1992). Activation of Insulin Receptor Signaling by a Single Amino Acid Substitution in the Transmembrane Domain. J. Biol. Chem..

[26] Li Q., Wong Y.L., Kang C. (2014). Solution Structure of the Transmembrane Domain of the Insulin Receptor in Detergent Micelles. Biochim. Biophys. Acta Biomembr..

[27] Mohammadiarani H., Vashisth H. (2016). All-Atom Structural Models of the Transmembrane Domains of Insulin and Type 1 Insulin-Like Growth Factor Receptors. Front. Endocrinol..

[28] Kuznetsov A.S., Zamaletdinov M.F., Bershatsky Y.V., Urban A.S., Bocharova O.V., Bennasroune A., Maurice P., Bocharov E.V., Efremov R.G. (2020). Dimeric States of Transmembrane Domains of Insulin and IGF-1R Receptors: Structures and Possible Role in Activation. Biochim. Biophys. Acta Biomembr..

[29] Kavran J.M., McCabe J.M., Byrne P.O., Connacher M.K., Wang Z., Ramek A., Sarabipour S., Shan Y., Shaw D.E., Hristova K. (2014). How IGF-1 Activates Its Receptor. eLife.

[30] Cavanagh J. (2007). Protein NMR Spectroscopy: Principles and Practice.

[31] Sattler M. (1999). Heteronuclear Multidimensional NMR Experiments for the Structure Determination of Proteins in Solution Employing Pulsed Field Gradients. Prog. Nucl. Magn. Reson. Spectrosc..

[32] Mielke S.P., Krishnan V.V. (2009). Characterization of Protein Secondary Structure from NMR Chemical Shifts. Prog. Nucl. Magn. Reson. Spectrosc..

[33] Güntert P. (2004). Automated NMR Structure Calculation with CYANA. Protein NMR Techniques.

[34] Chill J.H., Louis J.M., Baber J.L., Bax A. (2006). Measurement of 15N Relaxation in the Detergent-Solubilized Tetrameric KcsA Potassium Channel. J. Biomol. NMR.

[35] Shen Y., Bax A. (2013). Protein Backbone and Sidechain Torsion Angles Predicted from NMR Chemical Shifts Using Artificial Neural Networks. J. Biomol. NMR.

[36] Wagner G., Pardi A., Wuethrich K. (1983). Hydrogen Bond Length and Proton NMR Chemical Shifts in Proteins. J. Am. Chem. Soc..

[37] Hwang T.-L., van Zijl P.C.M., Mori S. (1998). Accurate Quantitation of Water-Amide Proton Exchange Rates Using the Phase-Modulated CLEAN Chemical EXchange (CLEANEX-PM) Approach with a Fast-HSQC (FHSQC) Detection Scheme. J. Biomol. NMR.

[38] Cybulski L.E., de Mendoza D. (2011). Bilayer Hydrophobic Thickness and Integral Membrane Protein Function. Curr. Protein Pept. Sci..

[39] Bocharov E.V., Mineev K.S., Pavlov K.V., Akimov S.A., Kuznetsov A.S., Efremov R.G., Arseniev A.S. (2017). Helix-Helix Interactions in Membrane Domains of Bitopic Proteins: Specificity and Role of Lipid Environment. Biochim. Biophys. Acta Biomembr..

[40] Bocharov E.V., Sharonov G.V., Bocharova O.V., Pavlov K.V. (2017). Conformational Transitions and Interactions Underlying the Function of Membrane Embedded Receptor Protein Kinases. Biochim. Biophys. Acta Biomembr..

[41] Polyansky A.A., Chugunov A.O., Volynsky P.E., Krylov N.A., Nolde D.E., Efremov R.G. (2014). PREDDIMER: A Web Server for Prediction of Transmembrane Helical Dimers. Bioinformatics.

[42] Efremov R.G., Alix A.J.P. (1993). Environmental Characteristics of Residues in Proteins: Three-Dimensional Molecular Hydrophobicity Potential Approach. J. Biomol. Struct. Dyn..

[43] Bugge K., Lindorff-Larsen K., Kragelund B.B. (2016). Understanding Single-Pass Transmembrane Receptor Signaling from a Structural Viewpoint—What Are We Missing?. FEBS J..

[44] Trenker R., Call M.J., Call M.E. (2016). Progress and Prospects for Structural Studies of Transmembrane Interactions in Single-Spanning Receptors. Curr. Opin. Struct. Biol..

[45] Polyansky A.A., Bocharov E.V., Velghe A.I., Kuznetsov A.S., Bocharova O.V., Urban A.S., Arseniev A.S., Zagrovic B., Demoulin J.-B., Efremov R.G. (2019). Atomistic Mechanism of the Constitutive Activation of PDGFRA via Its Transmembrane Domain. Biochim. Biophys. Acta Gen. Subj..

[46] Bocharov E.V., Bragin P.E., Pavlov K.V., Bocharova O.V., Mineev K.S., Polyansky A.A., Volynsky P.E., Efremov R.G., Arseniev A.S. (2017). The Conformation of the Epidermal Growth Factor Receptor Transmembrane Domain Dimer Dynamically Adapts to the Local Membrane Environment. Biochemistry.

[47] Arkhipov A., Shan Y., Das R., Endres N.F., Eastwood M.P., Wemmer D.E., Kuriyan J., Shaw D.E. (2013). Architecture and Membrane Interactions of the EGF Receptor. Cell.

[48] Volynsky P.E., Polyansky A.A., Fakhrutdinova G.N., Bocharov E.V., Efremov R.G. (2013). Role of Dimerization Efficiency of Transmembrane Domains in Activation of Fibroblast Growth Factor Receptor 3. J. Am. Chem. Soc..

[49] Martín-Segura A., Ahmed T., Casadomé-Perales Á., Palomares-Perez I., Palomer E., Kerstens A., Munck S., Balschun D., Dotti C.G. (2019). Age-Associated Cholesterol Reduction Triggers Brain Insulin Resistance by Facilitating Ligand-Independent Receptor Activation and Pathway Desensitization. Aging Cell.

[50] Delle Bovi R.J., Kim J., Suresh P., London E., Miller W.T. (2019). Sterol Structure Dependence of Insulin Receptor and Insulin-like Growth Factor 1 Receptor Activation. Biochim. Biophys. Acta Biomembr..

[51] Bocharova O.V., Urban A.S., Nadezhdin K.D., Bocharov E.V., Arseniev A.S. (2016). Cell-Free Expression of the APP Transmembrane Fragments with Alzheimer’s Disease Mutations Using Algal Amino Acid Mixture for Structural NMR Studies. Protein Expr. Purif..

[52] Schägger H., von Jagow G. (1987). Tricine-Sodium Dodecyl Sulfate-Polyacrylamide Gel Electrophoresis for the Separation of Proteins in the Range from 1 to 100 KDa. Anal. Biochem..

[53] Keller R. (2004). The Computer Aided Resonance Assignment Tutorial.

[54] Favier A., Brutscher B. (2011). Recovering Lost Magnetization: Polarization Enhancement in Biomolecular NMR. J. Biomol. NMR.

[55] Kazimierczuk K., Orekhov V.Y. (2012). A Comparison of Convex and Non-Convex Compressed Sensing Applied to Multidimensional NMR. A comparison of convex and non-convex compressed sensing applied to multidimensional NMR. J. Magn. Reson..

