# Supplementary material for: Diversity of Structural, Dynamic, and Environmental Effects Explain a Distinctive Functional Role of Transmembrane Domains in the Insulin Receptor Subfamily
Source: Int J Mol Sci. 2023 Feb 15;24(4):3906. doi: 10.3390/ijms24043906 (PMC9965288; doi:10.3390/ijms24043906)
Supplement: Supplementary file 1 [file ijms-24-03906-s001.zip › ijms-2168036-supplementary.pdf]

Table S1. Structural statistics for the ensemble of 20 best NMR structures of the InsRtm, IGF1Rtm and IRRtm monomers.

**NMR distance and dihedral restraints**	InsRtm	IGF1Rtm	IRRtm
Total unambiguous NOE restraints	274	274	204
Intraresidue	89	83	61
Inter-residue	185	191	143
Sequential, $\|i - j\| = 1$	62	60	42
Short-range, $\|i - j\| \leq 1$	151	143	103
Medium range, $1 < \|i - j\| \leq 4$	123	131	101
Long range, $\|i - j\| > 4$	0	0	0
Hydrogen bond restraints (upper/lower)	51/51	45/45	45/45
Total torsion angle restraints	57	71	56
Backbone φ	26	29	25
Backbone ψ	27	20	26
Side chain χ^1	4	12	5
**Structure calculation statistics**			
CYANA target function, Å^2	0.29 ± 0.09	0.56 ± 0.13	0.37 ± 0.12
Restraint violations			
Distance (>0.3 Å)	0	0	0
Dihedral (>5°)	0	0	0
Average pairwise RMSD[a], Å, overall TMD α-helical region			
Backbone atoms	0.51 ± 0.18	0.49 ± 0.19	2.19 ± 0.68
All heavy atoms	1.11 ± 0.20	1.06 ± 0.16	2.80 ± 0.75
Average pairwise RMSD[a], Å, stable α-helical region after intramembrane proline			
Backbone atoms	0.22 ± 0.10	0.24 ± 0.08	0.45 ± 0.17
All heavy atoms	0.66 ± 0.16	0.77 ± 0.21	1.06± 0.20
Ramachandran analysis[b]			
Residues in most favored regions, %	96.7	98.1	96.2
Residues in additional allowed regions, %	2.9	1.9	3.8
Residues in generously allowed regions, %	0.4	0.0	0.0
Residues in disallowed regions, %	0.0	0.0	0.0

[a] Root mean square deviation.
[b] Ramachandran statistics were determined using CYANA.

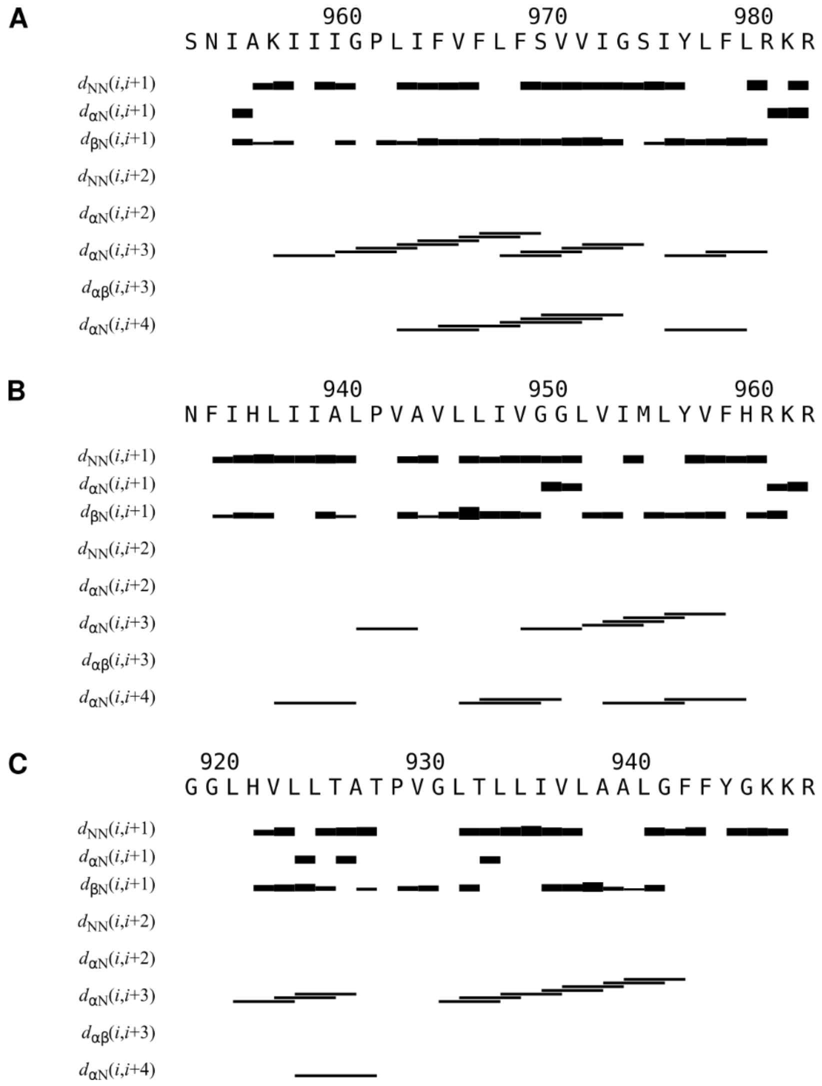

Figure S1. NMR data for the InsRtm, IGF1Rtm, and IRRtm monomers in a membrane-mimicking environment. Intramonomeric sequential and medium range NOE connectivity observed in 3D 15N-edited NOESY-HSQC spectra (80 ms mixing time) are shown for InsRtm (A), IGF1Rtm (B) and IRRtm (C), respectively, by horizontal lines. The line thickness for the NOE connectivity is inversely proportional to the squared upper distance bound. The NOE information on some regions was restricted due to cross-peak broadening and overlapping.
